# Surgical Management of Pericaecal Hernia in a Virgin Abdomen

**DOI:** 10.7759/cureus.56192

**Published:** 2024-03-14

**Authors:** Jia Ling Ong, Wei Chuan Tan, Kien Fatt Sean Lee, Kuan Yuen Yeong, Choon Sheong Seow

**Affiliations:** 1 Department of General Surgery, Ng Teng Fong General Hospital, Singapore, SGP; 2 Department of Radiology, Ng Teng Fong General Hospital, Singapore, SGP

**Keywords:** abdominal radiology, unexplained abdominal pain, paracaecal hernia, congenital transmesenteric hernia, virgin abdomen, closed loop obstruction, bowel ischemia, diagnostic laparoscopy, small intestinal obstruction, pericecal internal hernia

## Abstract

Internal hernia is an uncommon cause of mechanical small bowel obstruction. This case report details a 66-year-old Chinese male with no prior abdominal surgeries who presented with colicky abdominal pain, abdominal distension, and vomiting. Initial investigations were unyielding, but escalating symptoms prompted a diagnostic laparoscopy. Laparotomy then revealed a closed-loop obstruction through a lateral type pericecal hernia, with a segment of ischemic jejunum. Adhesion bands in the right iliac fossa and a congenital hernia orifice in the mesentery were identified and addressed. The patient recovered well postoperatively. This discussion explores the Meyer's classification of pericecal hernias, potential etiologies, clinical manifestations, diagnostic considerations, and the choice between laparoscopic and open surgeries. This case underscores the importance of a high index of suspicion, prompt surgical intervention, and the diagnostic utility of laparoscopy in managing pericecal hernias.

## Introduction

Internal hernias represent an infrequent etiology of small bowel obstruction [1]. Among these, pericecal hernias, alternatively termed paracecal hernias, stand out as an exceptionally uncommon subtype [2]. In the context of pericecal hernias, herniation typically occurs through an opening formed within the peritoneal recess, originating from the folds of the peritoneum in the paracecal region [3]. This case report documents an instance of pericecal hernia observed in a male with a virgin abdomen who developed a complication of bowel obstruction resulting in bowel ischemia.

## Case presentation

**Figure 1:**
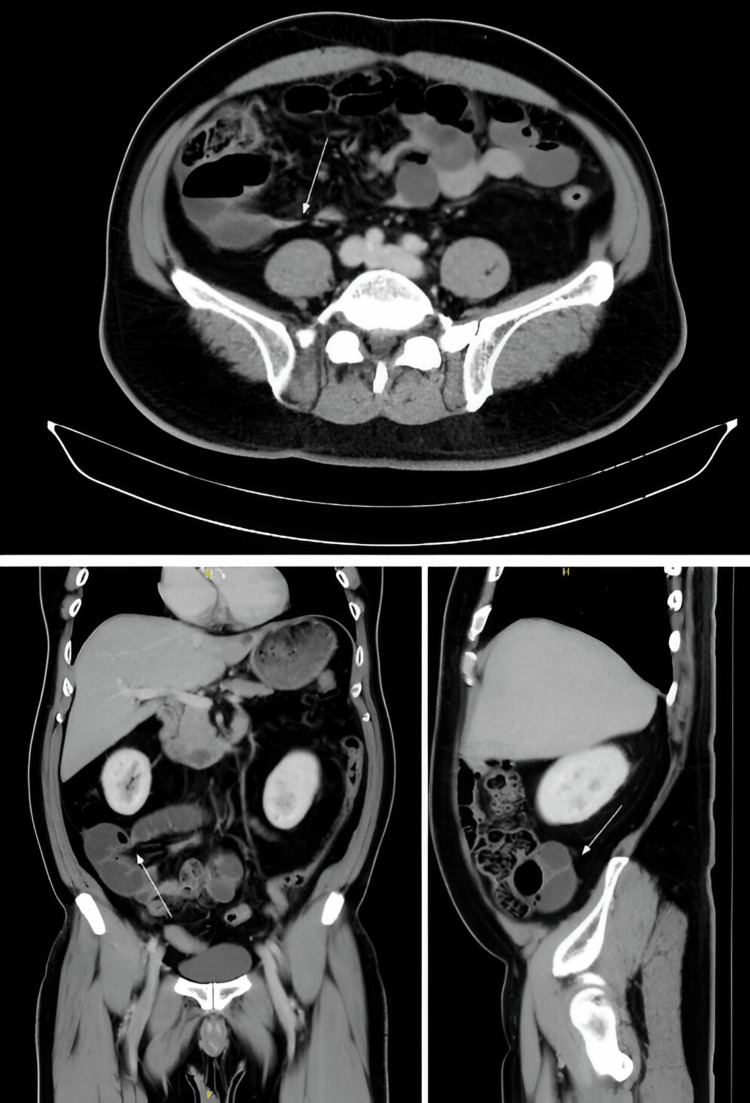
CT of the abdomen and pelvis. Axial and coronal images demonstrate sharp angulation of the small bowel loops (arrows) at the right iliac fossa possibly because of adhesion bands. Sagittal image demonstrates a loop of undilated small bowel posterior to the cecum (arrow) with preserved mural enhancement

A 66-year-old Chinese gentleman presented to our emergency department with colicky periumbilical pain radiating to the right lower abdomen for one day. He had a past medical history of hyperlipidemia and ischemic heart disease and was on aspirin. He had no previous abdominal surgeries. He underwent a colonoscopy last year, which revealed diverticula in the ascending and transverse colon, as well as a tubular adenomatous polyp with low-grade dysplasia in the ascending colon (which was removed). He had tenderness over the right lower abdomen without guarding or rebound tenderness. Laboratory investigations were unremarkable as his inflammatory markers and lactate levels were within normal limits. A preliminary contrasted computed tomography (CT) scan report did not show any abnormalities in the abdomen and pelvis, but, on retrospective review, there was a narrowing of the small bowel and possible adhesion band (Figure 1). He was allowed diet and given a fleet enema to clear his bowels.

**Figure 2:**
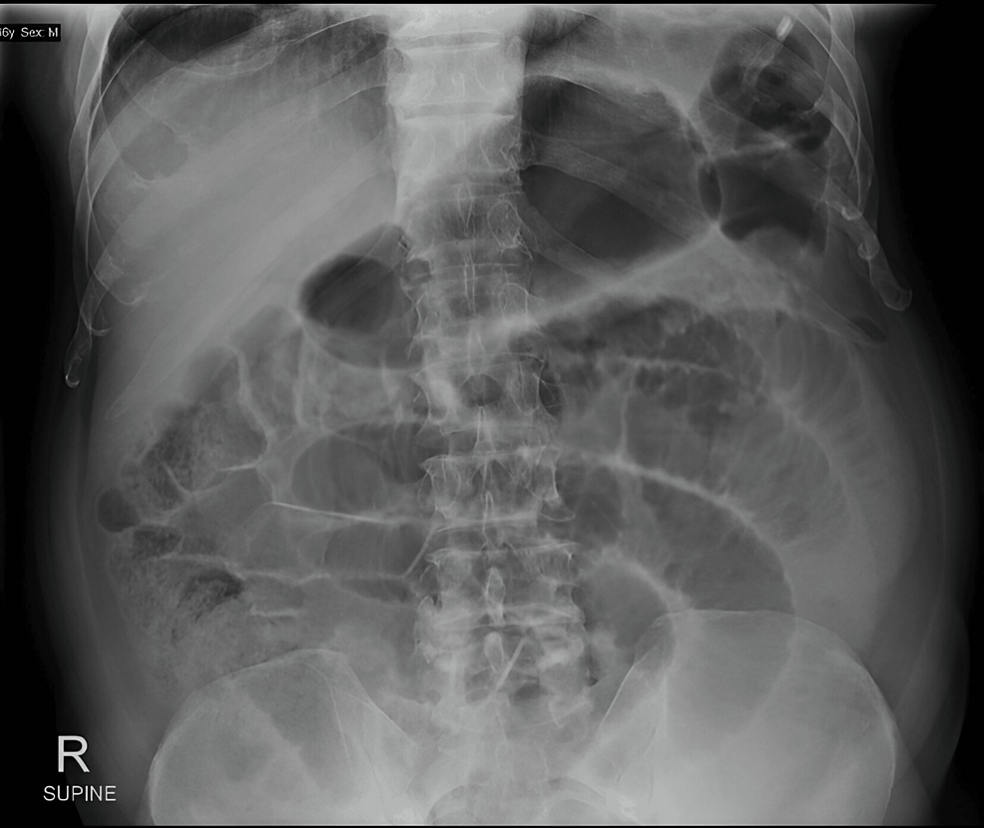
An abdominal X-ray revealed multiple dilated small bowel loops of up to 4.7cm in diameter with no evidence of intraperitoneal free gas

The following day, his symptoms persisted despite symptomatic treatment. Moreover, he expelled large amounts of vomit, and his abdomen became more distended. Tenderness over the right lower abdomen persisted but without signs of peritonism. He was kept fasted, and a nasogastric tube was inserted for decompression. Approximately 800 mL of bilious fluid was aspirated from his nasogastric tube, but his abdominal pain and distension did not improve. His total WBC count increased to 20,000/μL. An abdominal X-ray revealed multiple dilated small bowel loops up to 4.7 cm in diameter with no evidence of intraperitoneal free gas (Figure 2). These findings raised suspicion for intestinal obstruction. He was then counseled and underwent emergency diagnostic laparoscopy converted to laparotomy and small bowel resection.

**Figure 3:**
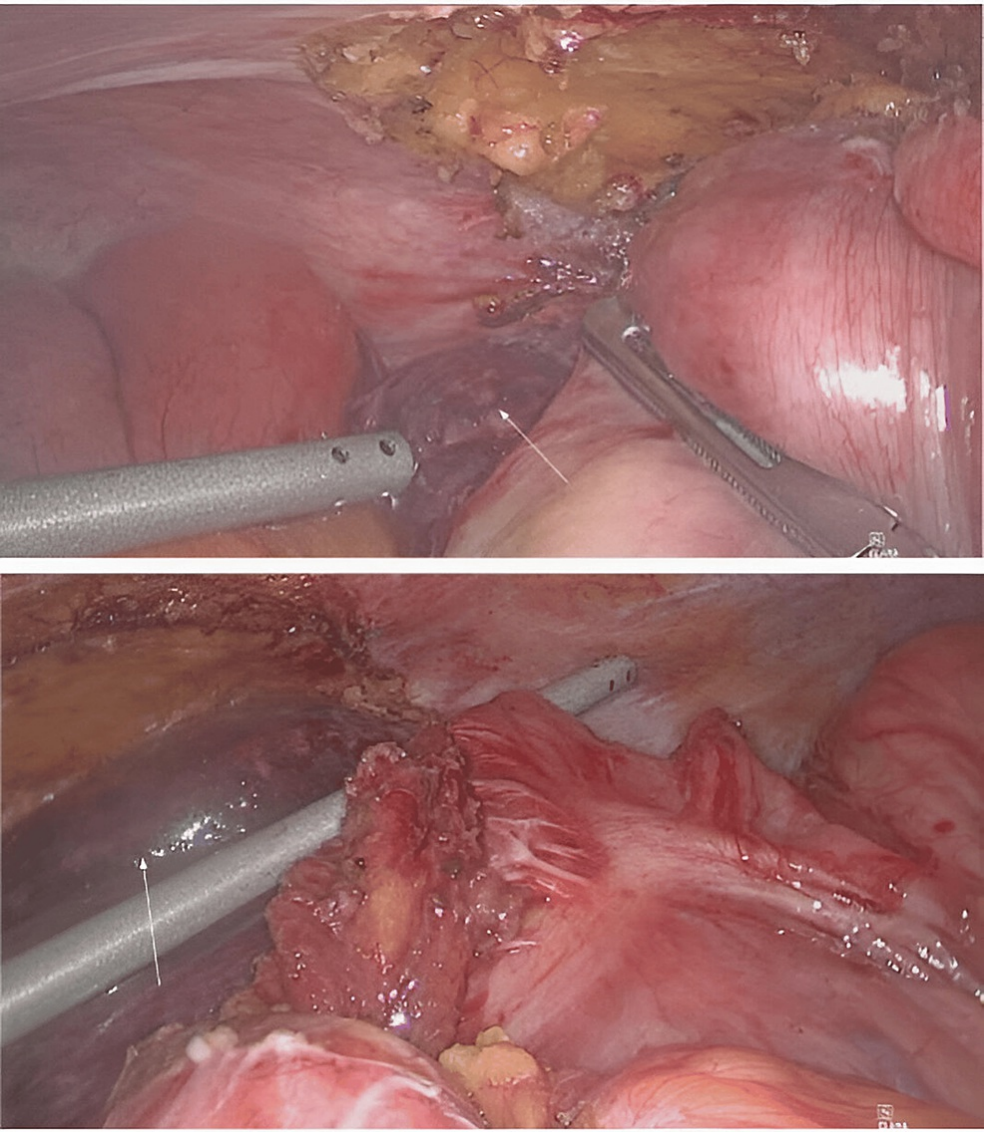
Intraoperative photo of the ischemic segment of jejunum during dissection. Arrows indicate the ischemic jejunal segment posterior to cecum

**Figure 4:**
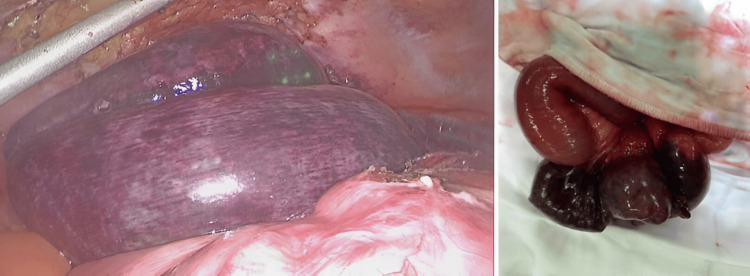
Intraoperative photo of the ischemic segment of jejunum after dissection

Intraoperative findings revealed closed-loop obstruction at the mid-jejunum resulting in a short segment of bowel ischemia. Adhesion bands in the right iliac fossa were divided. A segment of a nonviable jejunal loop was found behind the cecum, underneath a membranous layer of peritoneum (Figure 3). After laparoscopic mobilization, this segment of the ischemic jejunum was exteriorized via a mini-laparotomy (Figure 4). Small bowel resection was performed and a functional end-to-end stapler anastomosis was created. The rest of the small bowel was evaluated completely and found to be viable. A small internal hernia orifice in the cecal mesentery was identified and closed.

 

He had an uneventful postoperative recovery.

## Discussion

There are four subtypes of pericecal hernias: internal, retrocecal recess, lateral, and unclassifiable [4]. In our case, a loop of ischemic small bowel was found at the right paracolic gutter lateral to the ascending colon; hence, it can be classified as the lateral type. This loop of bowel possibly emerged through a congenital hernia orifice in the mesentery [5]. There were adhesion bands between the loop of the bowel and the lateral abdominal wall. Given that our patient has no previous abdominal surgeries, these adhesion bands are likely congenital. Another possible cause would be post-inflammatory changes from previous enterocolitis. 

Pericecal hernias can present with symptoms and signs of small bowel obstruction when the bowel is incarcerated. Symptoms include abdominal pain, nausea, vomiting, and constipation [6]. The clinical suspicion for small bowel obstruction should arise when the abdomen is distended and the suspicion for ischemia should be high when there is persistent abdominal tenderness. Laboratory tests such as inflammatory markers and lactate can assist in assessing the severity of the obstruction, but may not be deranged at initial presentation. Initial radiological investigations such as an abdominal X-ray can help in diagnosing small bowel obstruction, looking for dilated bowel loops and paucity of gas in the bowel. CT imaging is particularly useful in determining the etiology of the bowel obstruction, looking for complications, and assisting in pre-operative planning [7]. In retrospect, upon reviewing the coronal and sagittal images of the CT scans again, a small bowel transition point can be identified posterior to the cecum. This should raise the index of suspicion for pericecal hernias as the small bowels are usually medial to the large bowels and not posterior. The abnormal position of small bowel loops posterior to the cecal pole on the CT would raise the possibility of an underlying pericecal hernia. While this could be an incidental finding, the incidence and natural history of asymptomatic patients with internal hernia is not known. In a patient who shows signs and symptoms of intestinal obstruction, the presence of an internal hernia would warrant a prompt surgical review.

Urgent surgery is the recommended treatment for internal hernias, particularly when strangulation is suspected. The initial step involves reducing the hernia and assessing the bowel viability [8]. Subsequently, it is essential to address the hernia orifice by either opening or closing it to prevent recurrence. While laparoscopic surgery is gaining popularity for small bowel obstructions because of its high diagnostic accuracy and minimal invasiveness [9], we chose open laparotomy for our patient after confirming intestinal ischemia requiring bowel resection. The lack of adequate pneumoperitoneum given dilated proximal small bowels made it unsafe to attempt small bowel resection and anastomosis laparoscopically [10].

## Conclusions

Pericecal hernias represent a rare subtype of internal hernias. The consideration of strangulation becomes crucial in cases where clinical signs, laboratory results, and radiological findings are incongruent. Diagnostic laparoscopy emerges as a valuable diagnostic and therapeutic tool, particularly for patients experiencing small bowel obstruction with uncertain etiology.
